# A cross-sectional study of risk factors associated with sarcopenia in patients with metabolic dysfunction-associated steatotic liver disease

**DOI:** 10.3389/fmed.2025.1488068

**Published:** 2025-02-18

**Authors:** Johnny Amer, Qusay Abdoh, Zaina Salous, Eithar Abu Alsoud, Sama AbuBaker, Ahmad Salhab, Manal Badrasawi

**Affiliations:** ^1^Department of Allied Sciences, Faculty of Medicine and Health Sciences, An-Najah National University, Nablus, Palestine; ^2^Department of Internal Medicine, GI and Endoscopy Unit, An-Najah National University Hospital, Nablus, Palestine; ^3^Department of Medical Sciences, Faculty of Medicine and Health Sciences, An-Najah National University, Nablus, Palestine; ^4^Nutrition and Food Technology Department, An-Najah National University, Nablus, Palestine

**Keywords:** MASLD, sarcopenia, wSMI, handgrip strength, muscle mass

## Abstract

**Background:**

Metabolic dysfunction-associated steatotic liver disease (MASLD) is a chronic liver disease linked to several adverse health consequences that include metabolic disturbances affecting skeletal muscle. Sarcopenia, characterized by skeletal muscle loss, is commonly observed in individuals with MASLD. Our study aimed to identify modifiable lifestyle factors associated with sarcopenia in patients with MASLD.

**Methods:**

This study was a cross-sectional study that was conducted in three clinics in Nablus. A total of 162 adults diagnosed with MASLD were recruited for the study. The patients were interviewed and instructed to provide the necessary information, such as sociodemographic factors, medical and surgical history, lifestyle information, MASLD-related data, and nutritional and functional status. Sarcopenia was defined using the Foundation for the National Institutes of Health (FNIH) criteria, which includes the weight-adjusted skeletal muscle index (wSMI) with the cut-off scores (male subjects: 35.7% and female subjects: 30.7%). Statistical analysis was conducted using SPSS v.21. A chi-squared or independent samples t-test was utilized to identify the factors linked to sarcopenia in the study sample.

**Results:**

Our data found that 44% of MASLD patients had sarcopenia. This condition was significantly associated with female gender (*p* < 0.0001), older age (p < 0.0001), presence of chronic diseases (*p* < 0.035), and medication use (*p* < 0.05). Regarding nutritional factors, sarcopenia had a significant association with obesity, a higher body fat percentage, a high waist-to-hip ratio, a low mid-upper arm circumference, and a reduced calf circumference (*p* < 0.001). Sarcopenic individuals often exhibit reduced handgrip strength. Lifestyle factors such as a history of smoking and the type of smoking were found to be positively associated with sarcopenia (*p* < 0.0001).

**Conclusion:**

Sarcopenia was prevalent in the study population and was linked to modifiable risk factors that can be managed to reduce its progression. Future research using different study designs, such as longitudinal design, is recommended to identify the determinants of sarcopenia. Intervention studies are also required to improve the nutritional and functional status of MASLD patients.

## Introduction

Sarcopenia is a skeletal muscle condition characterized by a generalized, progressive loss of muscle strength and muscle mass that frequently occurs with aging (1, 2). However, it can also be influenced by factors such as inactivity, poor nutrition, chronic illnesses, and hormonal changes (3). Recently, the complex connection between sarcopenia and metabolic dysfunction-associated steatotic liver disease (MASLD)/metabolic dysfunction-associated steatohepatitis (MASH) has attracted academic attention.

MASLD is characterized by the presence of hepatic steatosis, which can be identified through imaging or histological methods, in individuals without significant alcohol consumption or other secondary causes of liver fat accumulation. The diagnosis of MASLD requires evidence of metabolic dysfunction, such as overweight or obesity, type 2 diabetes mellitus, or markers of metabolic dysregulation, including elevated waist circumference, hypertension, dyslipidemia, or insulin resistance (4). The most severe condition in this spectrum of MASLD is usually referred to as MASH. MASH is characterized by inflammation, liver damage, and fibrosis (scarring) that can lead to cirrhosis and liver failure if left untreated (5, 6).

The prevalence of MASLD has increased dramatically in Western countries, with a global prevalence of 25% (4). This rise is largely due to many risk factors that enhance the development of MASLD, such as obesity, hyperlipidemia, type 2 diabetes mellitus (T2DM), hypertension, and metabolic syndrome (6). The pathophysiology of MASLD development remains unclear.

Many published studies have identified a link between sarcopenia and the severity of MASLD as determined by liver histology (7, 8). The combination of high body mass index (BMI) and sarcopenia represents a unique and complex clinical phenotype, often referred to as sarcopenic obesity (7). It has been reported that patients with MASLD/MASH are prone to sarcopenia despite having a high BMI. The complication rate of sarcopenia ranges from 20.8 to 43.6%, and this rate tends to increase as fibrosis progresses (7, 8). The pathophysiology of high BMI combined with sarcopenia involves a complex interplay between metabolic, hormonal, and inflammatory factors that affect both the adipose tissue and skeletal muscle. In MASLD, sarcopenia is closely associated with insulin resistance and results from the atrophy of skeletal muscle, which is an insulin-target organ (7). The presence of sarcopenia is a prognostic factor and increases the risk of mortality in patients with cirrhosis and those who have undergone treatment for liver cancer (1).

According to previous research, some of these lifestyle behaviors that play a significant role are sleep duration, physical activity, and food selection, specifically the intake of nutrients such as protein and vitamin D (9–11). In addition, malnutrition contributes to the pathogenesis of sarcopenia (12).

This study aimed to investigate the lifestyle and nutritional status of patients diagnosed with MASLD. In addition, the study sought to examine the prevalence of sarcopenia and identify any modifiable risk factors linked with MASLD in the Palestinian population.

## Methods

### Study design and setting

This was a cross-sectional study. It was performed on MASLD patients who had documented liver biopsies and regularly attended healthcare facilities for subsequent monitoring and evaluation. The study was conducted from October 2023 to December 2023 at the following centers in Nablus City, West Bank, Palestine: An-Najah National Hospital, Al Rahma Medical Center, and Al Watani Hospital. Patients who agreed to participate in the study and signed the informed consent form were interviewed and asked to complete a structured questionnaire, undergo the InBody 120 scan, and perform a handgrip test. The collected data included sociodemographic characteristics, medical history, lifestyle, MASLD-related information, nutritional status, and sarcopenia-related data. The study included MASLD patients who met the inclusion criteria and were present at the specified centers during the data collection period. The inclusion criteria required participants to be adult patients with a confirmed diagnosis of MASLD. Patients were excluded if they had chronic liver disease, such as hepatic virus infection (hepatitis B surface antigen or hepatitis C antibody positive), drug-induced hepatic disease, Wilson’s disease, α1-antitrypsin deficiency, hemochromatosis, autoimmune liver disease, liver cirrhosis, or hepatocellular carcinoma. In addition, patients were excluded from the study if they had connective tissue diseases, hereditary disorders associated with obesity such as Prader–Willi syndrome, pathological obesity conditions such as Cushing syndrome, or made regular use of steatosis-inducing drugs such as steroids, amiodarone, or tamoxifen.

### Patient questionnaire

The study was conducted through interviews, and the participants were asked to complete a structured questionnaire. The questionnaire contained three sections (not shown). The first section focused on sociodemographic characteristics, such as age, gender, education level, and lifestyle-related data, such as smoking, physical activity, and sleep patterns. The second section included an assessment of nutritional status and anthropometric measurements, such as weight, height, BMI, waist circumference, and hip circumference. All measurements were taken following standard procedures using the InBody 120 tool. Dietary patterns were assessed based on adherence to the Mediterranean Diet (MD) recommended for MASLD patients. Adherence to the MD was evaluated using the validated Arabic version of the Mediterranean Diet Adherence Screener (MEDAS), which includes 14 items, with scores for high adherence, moderate adherence, and low adherence to the MD. In the third section, skeletal muscle functional status was assessed using the handgrip test, following the standard procedures outlined in the instrument manual. Skeletal muscle mass was assessed using the body composition analyzer InBody 120. Sarcopenia was defined according to the Foundation for the National Institutes of Health (FNIH) criteria based on muscle mass and strength. The cutoff points used are shown in the table below (13).

### Study tools and instruments

#### Dependent variables

According to the Foundation for the National Institutes of Health (FNIH), sarcopenia is a categorical variable defined by loss of skeletal muscle mass and weakness (13). Sarcopenia in female subjects is diagnosed when skeletal muscle mass is <30.7% of the body weight (weight skeletal muscle index (wSMI)) and handgrip strength is less than 20 kg. Sarcopenia in male subjects is diagnosed if skeletal muscle mass is <35.7% of the body weight (wSMI) and handgrip strength is less than 30 kg.

#### Independent variables

1. Sociodemographic variables: age: a continuous numerical variable; sex: male or female, a nominal categorical variable; area of residence: village, city, or camp, a nominal categorical variable; living status: with spouse, with family (parents), with relatives, or other, a nominal categorical variable; marital status: single, married, or other, a nominal categorical variable; occupation: working or not working, a nominal categorical variable; education level: primary school (up to 6th grade), secondary (7th–10th grade), high school (11th–12th grade), or university, an ordinal categorical variable; and number of family members: a numerical discrete variable.
2. Participants’ past medical, surgical, and medication history variables: having chronic diseases other than MASLD: yes or no, a nominal categorical variable; the number of chronic diseases: a quantitative discrete variable; having previous surgical history: yes or no, a nominal categorical variable; medication history: yes or no, a nominal categorical variable; polypharmacy (five or more pills): yes or no, a nominal categorical variable; and number of surgeries: a quantitative discrete variable.
3. Participants’ lifestyle variables: smoking: yes regular, irregular, and no, a nominal categorical variable; type of smoking: cigarettes, shisha, and both cigarettes and shisha, a nominal categorical variable; taking a nap: every day, most of the time (5–7 days/week), sometimes (3–4 days/week), a few times (1–2 days/week), never, a nominal categorical variable; duration of sleeping: a continuous numerical variable; perceived sleep problems: yes or no, a nominal categorical variable; physical activity category (IPAQ): low, moderate, and high, an ordinal variable; time to sleep: a continuous quantitative variable; time to wake up: a constant quantitative variable; nap duration: a continuous quantitative variable; and screen time: a continuous quantitative variable.
4. Nutritional status-related variables: waist-to-hip ratio (WHR): a numerical continuous variable; BMI: a numerical continuous variable; % of body fat: a numerical continuous variable; mid-upper arm circumference: a numerical continuous variable; calf circumference: a numerical continuous variable; handgrip: a numerical continuous variable; snacks per day: a numerical continuous variable; main meals per day: a numerical continuous variable; Mediterranean Diet adherence: low, moderate, and high, a categorical variable; nutritional counseling: yes or no, a nominal categorical variable; nutrition consultant: a dietitian, physician, or other healthcare provider who did not provide counseling, a nominal categorical variable; nutrition compliance: always, most of the time, sometimes, never, a nominal categorical variable; nutrition information: sourced from a dietitian, physician, or other healthcare provider or through search in books, brochures, and the Internet, a nominal categorical variable.
5. MASLD-related variables: duration of the disease (in months): a discrete quantitative variable; age at diagnosis (in years): a discrete quantitative variable; symptoms: no presenting symptoms or having presenting symptoms, a nominal categorical variable; MASLD medications: yes or no, a nominal categorical variable.

### Bias

To reduce selection bias, the participants were selected randomly from multiple centers using a structured questionnaire with simple, closed-ended questions. While using the InBody 120 tool, we asked all participants to remove any metallic items (coins, jewelry, etc.), take off their shoes and socks, and stand in a specific position described in the instrument manual. For optimal use of the handgrip test, we asked the patients to perform it twice, following the instructions provided in the manual.

### Study size

A total of 160 patients were included in the study using a convenience sampling method. The sample size was calculated using G power, with an alpha of 0.05 (two-sided) and 80% power, indicating that a minimum of 110 participants was needed to determine the prevalence of sarcopenia among MASLD patients. To determine the association between sarcopenia and lifestyle factors, the sample size was recalculated using a chi-squared test, with a 5% significance level and a power of 80%, resulting in a required sample size of 160 participants.

### Statistical methods

Data analysis was performed using SPSS v.21. Frequencies and percentages were obtained for each categorical variable. Normality was checked before performing inferential statistics. A non-parametric chi-squared test was used to investigate the relationship between dependent and independent variables. In addition, an independent samples *t*-test was used to examine the relationship between continuous and categorical variables.

### Ethical approval

Ethical approval for this study was obtained from the Institutional Review Board (IRB) of An-Najah National University (ANNU) (IRB serial number: stu.MRC-118-79-4-23). Participants were informed about the purpose, techniques, risks, and benefits of the study; and consent was obtained from all participants before their involvement. The confidentiality and privacy of the participants and their data were strictly maintained throughout the study, with access limited exclusively to the researchers and their supervisors.

## Results

### Participant recruitment

**Figure 1:**
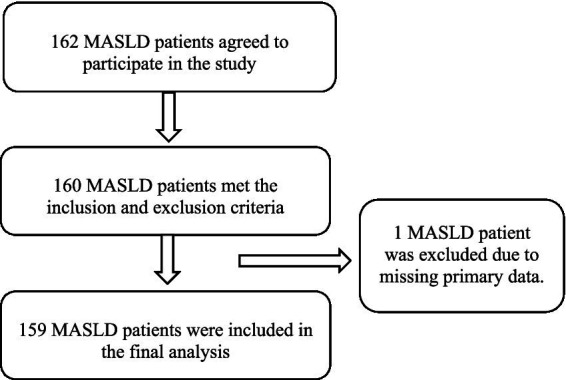
Steps in the recruitment of participants.

Participants were recruited from An-Najah National Hospital, Al Rahma Medical Center, and Al Watani Hospital, Nablus City, Northern Palestine. A total of 162 patients agreed to participate in the study. However, only 159 patients were included in the final analysis, as two did not meet the inclusion criteria and one was excluded due to missing primary data. A total of 57 participants (35.8%) were men and 102 participants (64.2%) were women—as shown in Figure 1.

### Demographic characteristics

**Figure 2:**
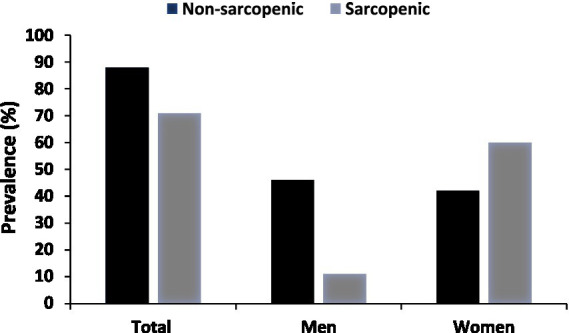
Prevalence of sarcopenia by gender, analyzed using a chi-squared test.

A total of 159 patients participated in the study, of whom 57 (35.8%) were men and 102 (64.2%) were women. Of these, 46.5% reported living in the city, the majority (82.4%) reported living with a spouse, 83.6% reported being married, 61.6% reported not working, and 35.8% reported having completed high school. The mean number of family members among participants was five, with a standard deviation of 2.31. Other related information is illustrated in Table 1. According to the analysis, we found a significant relationship between gender and sarcopenia, with a *p*-value of 0.0001. We also found a significant association between work and sarcopenia, with a *p*-value of 0.0001. There was also a significant relationship between age and sarcopenia, with a mean value of 50.8208 ± 11.97094 and a *p*-value of 0.001 (Figure 2).

**Table 1:** Sociodemographic characteristics of the participants.

Sociodemographic characteristics of the participants according to sarcopenia status are presented as numbers and (%)					
Variables		**Total**	**Normal**	**Sarcopenic**	***p*-value**
Gender	Male subjects	57 (35.8)	46 (80.7)	11 (19.3)	0.0001**
	Female subjects	102 (64.2)	42 (41.2)	60 (58.8)	
Area of residence	City	74 (46.5)	44 (59.5)	30 (40.5)	0.359
	Village	72 (45.3)	39 (54.2)	33 (45.8)	
	Camp	13 (8.2)	5 (38.5)	8 (61.5)	
Living status	Spouse	131 (82.4)	73 (55.7)	58 (44.3)	0.34
	With family	23 (14.5)	14 (60.9)	9 (39.1)	
	Other relatives	2 (1.3)	0 (0)	2 (100)	
	Other	3 (1.9)	1 (33.3)	2 (66.7)	
Marital status	Single	20 (12.6)	12 (60)	8 (40)	0.754
	Married	133 (83.6)	72 (54.1)	61 (45.9)	
	Other	6 (3.8)	4 (66.7)	2 (33.3)	
Occupation	Yes	61 (38.4)	46 (75.4)	15 (24.6)	0.000**
	No	98 (61.6)	42 (42.9)	56 (57.1)	
Education level	Primary school	17 (10.7)	9 (52.9)	8 (47.1)	0.302
	Secondary school	47 (29.6)	25 (53.2)	22 (46.8)	
	High school	57 (35.8)	28 (49.1)	29 (50.9)	
	University	38 (23.9)	26 (68.4)	12 (31.6)	
Age	43.9205	12.58737	50.8028	11.97094	0.001**
Number of family members	5.047	2.3106	5.000	2.5707	0.906

**p < 0.001.

### Participants’ medical, surgical, and medication history

A total of 159 patients with MASLD were included in the study. Nearly half of them (47.2%) reported having a chronic disease other than MASLD. Among them, 25.8% had one disease. The most common chronic condition among the patients with MASLD was hypertension (28.3%), followed by diabetes mellitus (18.2%). When the participants were asked about previous surgeries, 55.3% reported having had a prior surgery. Regarding regular medications, 49.1% reported using regular medications. Other related information is shown in Table 2.

**Table 2:** Medical, surgical, and medication history of the participants.

Medical, surgical, and medication history of the participants according to sarcopenia status are presented as number and (%)					
Variables		**Total**	**Normal**	**Sarcopenic**	***p*-value**
Chronic disease	Yes	75 (47.2)	35 (46.7)	40 (53.3)	0.038*
	No	84 (52.8)	53 (63.1)	31 (36.9)	
Previous surgery	Yes	88 (55.3)	47 (53.4)	41 (46.6)	0.584
	No	71 (44.7)	41 (57.7)	30 (42.3)	
Taking medication	Yes	78 (49.1)	37 (47.4)	41 (52.6)	0.049*
	No	81 (50.9)	51 (63)	30 (37)	
Polypharmacy (taking five or more pills)	Not taking medication	84 (52.8)	53 (63.1)	31 (36.9)	0.103
	Yes	3 (1.9)	1 (33.3)	2 (66.7)	
	No	72 (45.3)	34 (47.2)	38 (52.8)	

Medical, surgical, and medication history of the participants according to sarcopenia status are presented as Mean ± SD					
	**Normal**		**Sarcopenic**		***p*-value**
	**Mean**	**SD**	**Mean**	**SD**	
Number of chronic diseases	0.602	0.8649	0.887	1.0358	0.06
Number of previous surgeries	0.773	0.9313	1.056	1.5108	0.148

*p < 0.05.

### Descriptive data and outcomes of lifestyle-related variables

In the descriptive data statistics, the participants were categorized according to their lifestyle characteristics, as shown in Table 3. Among the participants, 29.6% smoked regularly, and 62.3% reported that they did not smoke. When asked about the type of smoking, 15.7% reported smoking cigarettes and 17% of them reported smoking shisha. Regarding the sleep data, 10.1% of all participants reported taking a nap every day, and 50% of them were reported as sarcopenic. Meanwhile, 52.2% of all participants reported not taking a nap, and 39.8% of them were reported as sarcopenic, with a mean nap duration (in minutes) among the sarcopenic participants with MASLD of 39.873 ± 53.8397. In addition, 41 participants in total had sleep problems, 36 of whom had insomnia. When we analyzed the lifestyle data, we found a highly significant association between sarcopenia and smoking history, with a *p*-value of 0.0001. Among the participants who were regular smokers, 23.4% were sarcopenic. Furthermore, there was a highly significant association between the type of smoking and sarcopenia, with a p-value of 0.0001, with 40.7% of shisha smokers being sarcopenic. All other related data are shown in detail in Table 3.

**Table 3:** Lifestyle-related variables.

Lifestyle characteristics of the participants according to sarcopenia status are presented as number and (%)					
Variables		**Total**	**Normal**	**Sarcopenic**	***p*-value**
Smoking	Yes, regular smoking	47 (29.6)	36 (76.6)	11 (23.4)	0.0001
	Yes, irregular smoking	13 (8.2)	9 (69.2)	4 (30.8)	
	No	99 (62.3)	43 (43.4)	56 (56.6)	
Type of smoking	Not a smoker	99 (62.3)	43 (43.4)	56 (56.6)	0.0001
	Cigarettes	25 (15.7)	23 (92)	2 (8)	
	Shisha	27 (17)	16 (59.3)	11 (40.7)	
	Both	8 (5)	6 (75)	2 (25)	
Taking a nap	Every day	16 (10.1)	8 (50)	8 (50)	0.765
	Most of the time (5–7 days/week)	13 (8.2)	7 (53.8)	6 (46.2)	
	Sometimes (3–4 days/week)	13 (8.2)	6 (46.2)	7 (53.8)	
	A few times (1–2 days/week)	34 (21.4)	17 (50)	17 (50)	
	Never	83 (52.2)	50 (60.2)	33 (39.8)	
Perceived sleep problems	Yes	41 (25.8)	20 (48.8)	21 (51.2)	0.186
	No	111 (69.8)	65 (58.6)	46 (41.4)	
Physical activity category (IPAQ)	Low	91 (57.2)	49 (53.8)	42 (46.2)	0.765
	Moderate	54 (34)	30 (55.6)	24 (44.4)	
	High	14 (8.8)	9 (64.3)	5 (35.7)	

Lifestyle characteristics of the participants according to sarcopenia status are presented as means (mean ± SD)					
Variable	**Normal**		**Sarcopenic**		***p*-value**
	**Mean**	**SD**	**Mean**	**SD**	
Sleep duration (in hours)	7.22	1.458	7.15	1.685	0.768
Sleep time	11:08:51.82	5:46:55.332	10:38:01.69	6:16:10.261	0.592
Wake up time	6:31:21.82	1:47:14.722	6:27:53.24	2:10:18.384	0.854
Nap duration (in minutes)	28.267	44.6657	39.873	53.8397	0.139
Screen time (in hours)	3.305	2.4428	3.092	2.1668	0.567

### Nutritional and functional status of the participants

When we analyzed the participants’ data on nutritional and functional status, we found a significant association between the participants’ BMI (mean of 3.9577 ± 0.2026), body fat percentage (mean of 47.2399 ± 7.07377), waist-to-hip ratio (mean of 1.0087 ± 0.03277), mid-upper arm circumference (mean of 35.57 ± 4.0571), calf circumference (mean of 40.758 ± 4.8483), and handgrip strength (mean of 13.0986 ± 11.80091), with a *p*-value of 0.001. However, no association was observed between the number of snacks and main meals per day. In addition, there were significant associations observed between nutrition consultations, with a *p*-value of 0.047. Specifically, 58.8% of the participants consulted a dietitian, 37.5% consulted a physician, 100% consulted other healthcare providers, and 39.3% did not receive counseling and were sarcopenic. There was also a significant relationship between nutrition compliance and sarcopenia, with a *p*-value of 0.017. Specifically, 87.5% of the participants were always compliant, 33.3% were primarily compliant, 56.3% were sometimes compliant, 39% did not receive counseling, and 69.2% were never compliant and sarcopenic. Finally, there was a significant association between sources of nutrition information and sarcopenia, with a p-value of 0.037. Specifically, 49.3% of the participants preferred to take information from dietitians, 36.1% from physicians, 100% from other healthcare providers, and 100% from the Internet, books, and other sources and were sarcopenic (Table 4).

**Table 4:** Nutritional and Functional Status.

Nutritional and functional status of the participants according to sarcopenia status presented as mean ± SD					
Variables	**Normal**		**Sarcopenic**		***p*-value**
	**Mean**	**SD**	**Mean**	**SD**	
BMI	3.3523	0.54751	3.9577	0.2026	0.0001
% of body fat	34.1483	6.4262	47.2399	7.07377	0.0001
Waist: Hip ratio	0.9589	0.03367	1.0087	0.03277	0.0001
MUAC	33.232	3.5047	35.57	4.0571	0.0001
Calf C	37.92	3.5183	40.758	4.8483	0.0001
Handgrip	26.4318	16.73649	13.0986	11.80091	0.0001
Snacks per day	2.093	1.2236	1.93	1.0327	0.373
Main meals per day	2.136	0.6099	1.972	0.6088	0.093

The nutritional and functional status of the participants according to sarcopenia status are presented as number and (%)					
		**Total**	**Normal**	**Sarcopenic**	***p*-value**
MEDAS	Low	9 (5.7)	4 (44.4)	5 (55.6)	0.578
	Moderate	104 (65.4)	56 (53.8)	48 (46.2)	
	High	46 (28.9)	28 (60.9)	18 (39.1)	
Nutrition counseling	Yes	42 (26.9)	19 (45.2)	23 (54.8)	0.132
	No	114 (73.1)	67 (58.8)	47 (41.2)	
Nutrition consultant	Dietitian	34 (21.7)	14 (41.2)	20 (58.8)	0.047
	Physician	8 (5.1)	5 (62.5)	3 (37.5)	
	Other healthcare provider	3 (1.9)	0 (0.0)	3 (100)	
	Did not receive counseling	112 (71.3)	68 (60.7)	44 (39.3)	
Nutrition compliance	Always	8 (5.1)	1 (12.5)	7 (87.5)	0.017
	Mostly	15 (9.6)	10 (66.7)	5 (33.3)	
	Sometimes	16 (10.2)	7 (43.8)	9 (56.3)	
	Did not receive counseling	105 (66.9)	64 (61)	41 (39)	
	Never	13 (8.3)	4 (30.8)	9 (69.2)	
Nutrition information	Dietitian	75 (47.5)	38 (50.7)	37 (49.3)	0.037
	Physician	36 (22.8)	23 (63.9)	13 (36.1)	
	Other healthcare providers	5 (3.2)	0 (0.0)	5 (100)	
	Searched books and brochures	1 (0.6)	0 (0.0)	1 (100)	
	The Internet	41 (25.9)	26 (63.4)	15 (36.6)	

### Descriptive data and outcomes of MASLD-related variables

The results of the disease-related variables are presented in Table 5. The analysis showed a highly significant association between the age of MASLD diagnosis and sarcopenia (*p* < 0.01), with a mean of 49.634 ± 11.241 among the sarcopenic participants compared to a mean of 42.273 ± 13.5509 in the regular participants.

**Table 5:** MASLD-related variables.

MASLD-related characteristics of the participants according to sarcopenia status are presented as means (mean ± SD)					
Variable	**Normal**		**Sarcopenic**		***p*-value**
	**Mean**	**SD**	**Mean**	**SD**	
Disease duration (in months)	19.841	30.4819	22.029	37.0374	0.684
Age at diagnosis	42.273	13.5509	49.634	11.2418	0.0001

MASLD-related characteristics of the participants according to sarcopenia status are presented as n (%)					
Variables		**Total**	**Normal**	**Sarcopenic**	***p*-value**
Symptoms	No presenting symptoms	144 (90.6)	80 (55.6)	64 (44.4)	0.540
	Presenting symptoms	15 (9.4)	8 (53.3)	7 (46.7)	
MASLD medication	Yes	16 (10.1)	6 (37.5)	10 (62.5)	0.104
	No	139 (87.4)	80 (57.6)	59 (42.4)	

## Discussion

In recent years, clinical data have revealed the coexistence of MASH/MASLD and sarcopenia. Studies have indicated that the presence of sarcopenia increases the risk of MASLD by more than fivefold, highlighting the critical role of muscle health in liver and metabolic function. This association suggests that patients with sarcopenia, even those without traditional risk factors such as obesity or metabolic syndrome, may benefit from regular screening for MASLD [46, 69]. In a cross-sectional study published in 2023 in the Republic of Korea, the prevalence of sarcopenia in non-obese patients with MASLD was 31.8% (approximately 30% in men and 40% in women, with a *p*-value <0.001), using the definition of sarcopenia based on body mass index-adjusted skeletal muscle area (SMA) (14). Almeida NS et al. showed that among 1,351 patients with MASLD, 17.7% were sarcopenic, defined by appendicular lean mass divided by BMI. They tended to be older women with worse metabolic status, which is consistent with the findings of our study (15). However, we found that there are different criteria for defining sarcopenia, which may lead to differences in prevalence rates across studies. This is consistent with an article published in 2022 that studied the prevalence of sarcopenia using different methods in patients with MASLD (15).

The findings of the study revealed a correlation between sarcopenia and sociodemographic factors, including gender, employment status, and age. Notably, the prevalence of sarcopenia was found to be higher in female patients, aligning with previous research indicating a higher prevalence of sarcopenia in female individuals compared to male individuals with MASLD (16, 17). This association suggests that women lose muscle faster than men because their hormones change more quickly, especially between the ages of 65 and 74, and that women’s levels of sex hormones, such as estrogen and androgens, drop more rapidly than men’s (18). This finding is inconsistent with that of previous research indicating a greater prevalence of sarcopenia in men compared to women with MASLD (19–21). This association suggests the potential role of androgens in maintaining muscle mass, with declining androgen levels in aging men possibly contributing to the increased prevalence of sarcopenia. Moreover, older women tend to engage more in physical activities such as housework, shopping, and square dancing, all of which are known to promote muscle mass (20). This suggests that employed MASLD patients may have a lower prevalence of sarcopenia compared to their unemployed counterparts, possibly due to the muscle-building effects of physical activity associated with work.

The findings of the study revealed a notable statistical connection between age and the prevalence of sarcopenia in MASLD patients. This discovery aligns with the findings of previous research (20, 21) indicating a significant association between age and sarcopenia in individuals with MASLD. As supported by the existing literature, this correlation may stem from the increased susceptibility to MASLD and the loss of muscle mass often associated with aging (20). According to the findings of the study, sarcopenia displayed a correlation with both medical history and medication usage. The present investigation revealed a higher prevalence of sarcopenia in MASLD patients who had chronic diseases. This outcome mirrors findings in previous studies indicating a heightened prevalence of diabetes mellitus, hypertension, elevated blood pressure, and increased serum levels of various biomarkers in individuals with sarcopenia (21). Furthermore, another study demonstrated a significant association between MASLD, sarcopenia, and an increased risk of ASCVD in the general population (22). The diagnosis of sarcopenia in patients with chronic diseases is considered to guide and tailor treatment in several ways, as muscle mass and function are closely linked to metabolic health and disease progression. There is growing evidence suggesting that therapies targeting metabolic dysfunction (e.g., insulin resistance) may also benefit muscle mass preservation. For instance, GLP-1 receptor agonists (such as semaglutide) or SGLT2 inhibitors are being studied for their effects on both liver fat reduction and muscle preservation (23). Managing sarcopenia through exercise prescription, nutrition and protein intake optimization, and administration of vitamin D and other micronutrients may also be particularly beneficial for sarcopenic patients (24).

According to the study findings, sarcopenia in MASLD patients was strongly associated with certain lifestyle-related characteristics, such as smoking and the type of smoking, both of which had a *p*-value of <0.01. This finding is in line with that of a previous study (11), which was a review confirming that smoking has a direct association with sarcopenia and lower skeletal muscle mass. It is also associated with poor lifestyle behaviors, such as inadequate nutrition and physical inactivity, which increase the risk of developing sarcopenia. In terms of nutritional and functional status, sarcopenia was significantly associated with BMI, body fat percentage, waist-to-hip ratio, mid-upper arm circumference, and calf circumference, with a *p*-value of 0.0001. In addition, nutrition consultants, sources of nutrition information, and compliance were significantly associated with sarcopenia, with a *p*-value <0.048. This is consistent with recent data showing the association of obesity and nutritional status with sarcopenia (7, 8, 11). In addition, there was a significant association between handgrip strength (mean of 13.0986 ± 11.80091) and sarcopenia, with a *p*-value of 0.0001, as described in a previous study (15). There was a highly significant association between sarcopenia and the age at diagnosis of MASLD, with sarcopenic patients having a higher mean age of 49.63 ± 11.24 compared to non-sarcopenic patients, whose mean age was 42.27 ± 13.55. Previous studies have explained this finding by noting that sarcopenia is more common in older adults as aging is associated with metabolic changes, a decrease in activity, and an inflammatory process, in addition to the inflammatory effect of MASLD, which increases the risk of developing sarcopenia (7). The study also suggests that the treatment plan for sarcopenia may need to be adapted based on gender due to physiological differences between men and women. While the underlying principles of sarcopenia treatment—exercise, nutrition, and sometimes medication—are similar for both genders, specific strategies may vary based on biological differences in muscle mass, hormonal influences, and other factors.

It is worth mentioning this study’s limitations as it is a cross-sectional work, which could show a relationship between the factors, but it cannot indicate a cause-and-effect relationship between these variables. In addition, there could be misreporting and recall bias, as a significant part of the data relied on the patients’ responses to the questionnaire. In addition, during our data analysis, we utilized cutoff points for sarcopenia from various countries, as specific cutoff points for Palestine were unavailable.

## Data Availability

The raw data supporting the conclusions of this article will be made available by the authors, without undue reservation.
